# The first complete plastome of *Mimusops coriacea* (A. DC.) Miq. (Sapotaceae)

**DOI:** 10.1590/1678-4685-GMB-2021-0174

**Published:** 2022-01-24

**Authors:** Rafaela Jorge Trad, Saura Rodrigues da Silva, Maria do Carmo Estanislau do Amaral

**Affiliations:** 1 Universidade Estadual de Campinas (UNICAMP), Instituto de Biologia, Departamento de Biologia Vegetal, Campinas, SP, Brazil.; 2 Universidade Federal de Minas Gerais (UFMG), Instituto de Ciências Biológicas (ICB), Laboratório de Macroecologia, Belo Horizonte, MG, Brazil.; 3 Universidade Estadual Paulista (UNESP), Faculdade de Ciências Agrárias e Veterinárias, Departamento de Biotecnologia Agropecuária e Ambiental, Jaboticabal, SP, Brasil.

**Keywords:** Ericales, complete chloroplast genome, Sapotoideae

## Abstract

*Mimusops coriacea* (A. DC.) Miq. (Sapotoideae, Sapotaceae, Ericales) is native to Madagascar and the Comoro Islands. This species is cultivated in many countries around the world and grows on sand in coastal vegetation. Here we sequenced, assembled, and annotated the first complete chloroplast genome of *M. coriacea*. The newly assembled chloroplast was analyzed with other available chloroplasts of Sapotaceae. Our results found a general conserved structure. The complete chloroplast genome has 159,689 bp, including 133 genes distributed in four regions: a large single-copy region of 88,887 bp, a small single-copy region of 18,618 bp, and two inverted repeats of 26,092 bp each. Our maximum likelihood phylogenetic tree was generated with 80 protein-coding genes and recovered a monophyletic Sapotaceae sister to a clade formed by Ebenaceae + Primulaceae. In our analysis, *Mimusops coriacea* clustered with the other eight species of Sapotaceae included in the study.

The family Sapotaceae (Ericales) is represented by 53 genera and between 1,100 and 1,275 species (Stevens, 2001, onwards). In Brazil, there are 13 genera, including about 240 species, which grow mostly in the Amazon and the Atlantic Forest (Alves-Araújo et al., 2020). Although the genus *Mimusops* L. has 41 species only two occur in Brazil, both cultivated: *Mimusops coriacea* (A. DC.) Miq. and *M. elengi* L. (Pennington, 1990; Alves-Araújo, 2020). *Mimusops coriacea* is native only to Madagascar and to the Comoro Islands, where it grows on the sand in coastal forests and its edible fruits can be found in local markets (Gautier et al., 2012). This species has also been widely cultivated in the tropics. The flowers of *M. coriacea* are similar to those of other species in the genus and are among the most complex in the family. They have two whorls of sepals, corolla lobes with lateral appendages, and staminodes (Pennington, 1990; Gautier *et al.,* 2012). The wood of *Mimusops coriacea* is used for construction, its branches and branchlets are fermented to prepare a beverage in the Antilles, and the fruits can be eaten; it is also used in traditional medicine in Ecuador (Bustamante et al., 2019), and recently its phytochemical and bioactive potential was corroborated (Bustamante *et al.,* 2021). According to Pennington (1990), the identification of genera of Sapotaceae is challenging and depends on a combination of characters, since no character is diagnostic. Quite different treatments were proposed depending on the character chosen (compare Aubreville (1964) and Baehni (1965) as an example). This situation, added to the absence of a comprehensive treatment for the family in the Neotropics since *Flora Brasiliensis* (Miquel, 1863), resulted in considerable confusion with ill-defined generic limits and genera with numerous synonyms (Pennington, 1990). Thus, the development of tools that allow us to address phylogenetic relationships and clarify generic limits is extremely important for Sapotaceae. To our knowledge, there are published plastomes from only six genera in the family (Jo et al., 2016, Khayi et al., 2020, Liu et al., 2019, Niu et al., 2018, 2020, Tao et al., 2021, Wang et al., 2021). Therefore, we present the first complete plastome for a species of the genus *Mimusops*.

Leaves from the collection N. Hanazaki n. 33759 deposited in the herbarium at the University of Campinas (UEC) under the voucher number 90949 were used for DNA extraction. The leaves were grinded for 60 seconds in 2 mL tubes using TissueLyserII (Qiagen, Hilden, Germany - Cat. No. 85300) and the total DNA was extracted using a modified version of the Doyle and Doyle (1987) CTAB protocol. The DNA was purified using the Wizard® SV Gel and PCR Clean-Up System (Promega, Madison, USA - Cat. No. A9282). Whole-genome libraries were prepared by Genohub Inc. facility (Austin, USA) and paired-end reads (2 x 150 bp) were sequenced on Illumina NextSeq 500 platform (Illumina Inc., San Diego, USA), resulting in a total of 10,687,184 reads. The whole chloroplast was *de novo* assembled using NOVOPlasty v. 3.8.2 (Dierckxsens et al., 2017) using the *rbc*L, accession number L01932.2, as seed. Reads were mapped to the chloroplast with Bowtie2 (Langmead and Salzberg, 2012) plugin in Geneious 9 (Kearse et al., 2012) using default parameters to validate the assembly and verify the coverage. A total of 503,543 reads mapped the assembled plastome with mean coverage of 412.8. Automatic annotation was done using GeSeq (Tillich et al., 2017) implemented on the Chlorobox website. Start and stop codons were visually inspected and manually adjusted on Geneious 9 (Kearse *et al.,* 2012), and tRNAs limits were corrected based on ARAGORN (Laslett and Canback, 2004) output. Potential pseudogenes were defined by Blast following Silva et al. (2018). The annotated chloroplast was submitted to GenBank under the accession number MW846242. A circular map was generated with OGDRAW (Lohse et al., 2013). Five plastomes from Sapotaceae had the quadripartite structure annotated, i.e., the large single copy (LSC), the small single copy (SSC), and the two inverted repeats (IR): *Madhuca hainanensis* Chun & F.C. How, *Manilkara zapota* (L.) P. Royen, *Pouteria caimito* (Ruiz & Pav.) Radlk., *Sideroxylon wightianum* Wall. and *Synsepalum dulcificum* (Schumach. & Thonn.) Daniell. These five plastomes were aligned with *Mimusops coriacea* using progressive Mauve algorithm in Mauve Plugin v. 2.3.2 (Darling et al., 2004) in Geneious 9 (Kearse *et al.,* 2012) to check for structural differences such as inversions or rearrangements. The limits between the four main regions of these six plastomes were evaluated with IRscope (Amiryousefi et al., 2018). Forward, palindromic, complementary, and reverse repeats were identified in REPuter (Kurtz et al., 2001); parameters were set as follows: minimal size of 30 bp and Hamming distance of 3. Simple sequence repeats (SSRs) were identified in MISA (Beier et al., 2017; Thiel et al., 2003) with a minimum number of 7, 4, 4, 3, 3, and 3, for mono-, di-, tri-, tetra-, penta-, and hexanucleotide repeats, respectively. To determine the position of *Mimusops coriacea* within Sapotaceae we selected 16 plastomes from Ericales on the GenBank database: three from Ebenaceae, two from Primulaceae, eight from Sapotaceae, two from Styracaceae, and one from Theaceae. All species information and GenBank accession numbers are presented in Table S1. A total of 80 protein-coding and four rRNA genes were aligned with MAFFT v 7.435 (Katoh and Standley, 2013). A maximum likelihood tree based on the concatenation approach considering different evolutionary models for each locus was generated with IQ-TREE 2 (Minh et al., 2020).

The general structure and the gene order of the plastome of *M. coriacea* are similar to those of the other five genera of Sapotaceae (*Madhuca hainanensis*, *Manilkara zapota*, *Pouteria caimito*, *Sideroxylon wightianum* and *Synsepalum dulcificum*) (Figure 1). The complete plastome has a total length of 159,689 bp, including a LSC of 88,887 bp, a SSC of 18,618 bp, and two IRs each of 26,092 bp. The GC content was 36.8% for the complete chloroplast, 34.6% for the LSC, 30.2% for the SSC and 42.9% for the IRs. The whole plastome and its four main regions have both similar sizes and respective GC content among all analyzed Sapotaceae species (Table 1). *Mimusops coriacea* plastome contains a total of 133 genes of which 81 are unique protein-coding genes, 30 are tRNA and four are rRNA genes (Figure 2). Seven protein-coding, seven tRNA and four rRNA genes are in the IR duplicated region. No rearrangements were observed in the chloroplast of the analyzed Sapotaceae species as occur in some other families from Ericales. However, rearrangements have been reported in the order, i.e., the *clp*P loss in Actinidiaceae (Wang et al., 2016), the highly reduced and rearranged plastome of non-photosynthetic Ericaceae (Logacheva et al., 2016; Braukmann et al., 2017), a 20 kb inversion in two genera of Styracaceae (Cai et al., 2021), and the pseudogenization of the pt-*accD* and transfer to the nucleus in *Primula sinensis* Sabine ex Lindle (Primulaceae) (Liu et al., 2016).

**Figure 1 ‒:**
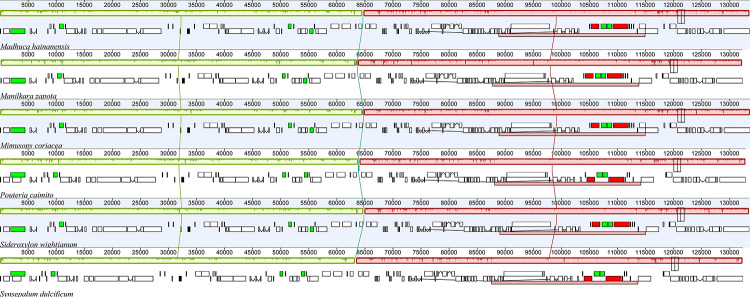
Progressive Mauve alignment showing synteny and rearrangements for six Sapotaceae plastomes. Species names are indicated below each respective plastome.

**Figure 2 ‒:**
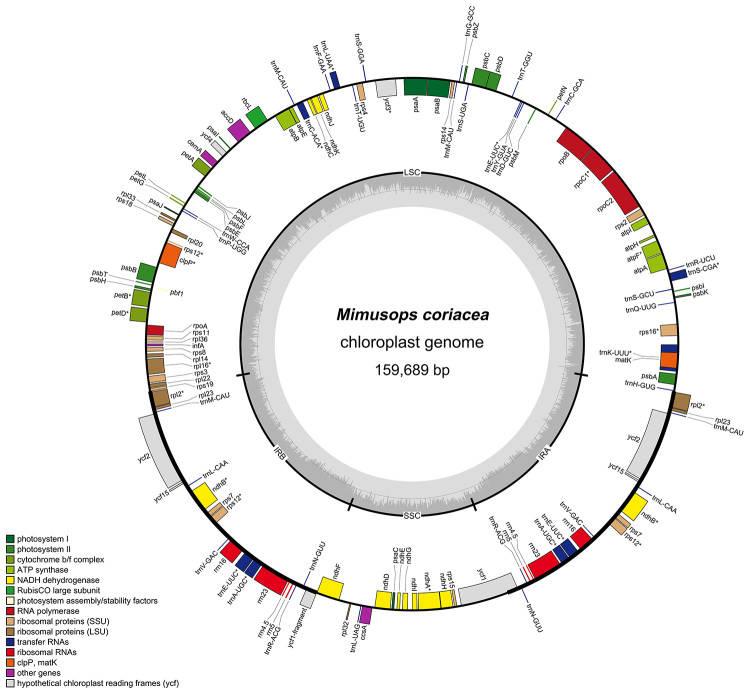
Circular map of *Mimusops coriacea* complete chloroplast genome. The genes represented outside the circle are transcribed counterclockwise and those inside the outer circle are transcribed clockwise. Genes are colored according to their functional groups following the legend. The inner gray graphs indicate the GC content across the plastome.

**Table 1 ‒:** Comparison of chloroplast genome size and GC content across three different regions (LSC, SSC, and IR) for six Sapotaceae species. LSC - large single copy; SSC - small single copy; IR - inverted repeat.

Species	LSC		SSC		IR		Complete plastome	
	bp	% GC	bp	% GC	bp	% GC	bp	% GC
*Madhuca hainanensis* Chun & F.C. How	88,846	34.6	18,598	30.1	26,093	42.9	159,630	36.8
*Manilkara zapota* (L.) P.Royen	87,745	34.9	18,443	30.2	26,099	42.9	158,386	37.0
*Mimusops coriacea* (A. DC.) Miq.	88,887	34.6	18,618	30.2	26,092	42.9	159,689	36.8
*Pouteria caimito* Radlk.	88,096	34.6	18,620	30.2	26,100	42.9	158,916	36.8
*Sideroxylon wightianum* Hook. & Arn.	89,043	34.7	18,365	30.4	26,065	42.9	159,538	36.9
*Synsepalum dulcificum* (Schumach. & Thonn.) Daniell	87,574	34.7	18,635	30.1	26,127	42.9	158,463	36.9

All the analyzed Sapotaceae plastomes have the junctions between single-copy and repeated regions in the same location as those of a typical angiosperm plastome (Wang et al., 2018). The LSC-IRb limit is flanked by *rpl*22 and *rps*19 on the LSC side and by *rpl*2 on the IRb side. The IRb-SSC junction has the *ycf*1-fragment on IRb in *Madhuca hanainensis* and *Mimusops coriacea* and the *ndh*F on SSC; only in *Synsepalum dulcificum* the *ndh*F spans the junction. We believe the absence of the *ycf*1-fragment in other species is just an annotation issue. The *ycf*1 spans the SSC-IRa junction and the IRa-LSC junction is flanked by *rpl*2 on IRa side and by *trn*H on LSC in all species but *Synsepalum dulcificum* which has a tRNA instead of the *trn*H (Figure S1). In *Mimusops coriacea* chloroplast, 16 genes have one intron each and two genes (*ycf*3 and *clp*P) have two introns each. REPuter (Kurtz et al., 2001) identified 49 long repeats in *Mimusops coriacea*, being 15 palindromic, 18 forward, eight reverse and eight complementary. For the other five Sapotaceae species included, between 30 repeats in *Synsepalum dulcificum* and 50 repeats in both *Madhuca hainanensis* and *Sideroxylon wightianum* were found (Table S2). MISA v. 2.1 (Beier et al., 2017; Thiel et al., 2003) identified 344 SSRs throughout *Mimusops coriacea* plastome. Among these repeats 287 are mono-SSR and 47 are di-SSR, four are tri-SSR, five are tetra-SSR and one is penta-SSR. Similar number of repeats were observed in the other five Sapotaceae species included (Table S2).

Relationships within Ericales are not fully understood (Stevens, 2001, onwards). In our maximum likelihood tree, Sapotaceae was recovered as monophyletic and sister to the clade Ebenaceae + Primulaceae, which agrees with Rose *et al.* (2018). On the other hand, Larson et al. (2020) recovered Sapotaceae as sister to Ebenaceae, and Primulaceae not close to that clade. In Sapotaceae, *Mimusops coriacea* clustered with the other eight species from the family (Figure 3). Our tree also showed a paraphyletic *Pouteria* Aubl. The complete chloroplast genome of *Mimusops coriacea* may contribute to future evolutionary studies within Sapotaceae, including a better comprehension of generic limits and interspecific relationships.

**Figure 3 ‒:**
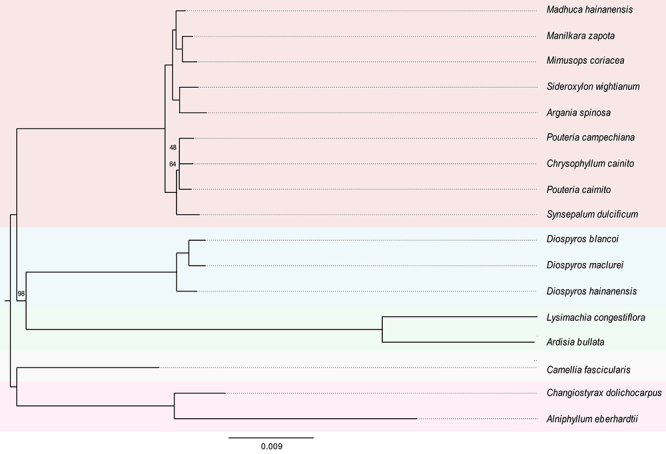
Phylogenetic tree of 16 Ericales species based on 80 chloroplast protein-coding genes and four rRNAs generated by maximum likelihood method. Numbers above branches represent ultrafast bootstrap support values; nodes with support lower than 100% are indicated. Theaceae and Styracaceae were used as outgroups. The background was colored as follows: Sapotaceae - red, Ebenaceae - blue, Primulaceae - green, Theaceae - gray, Styracaceae - pink.

## Supplementary material

The following online material is available for this article:

Table S1 - Species names, family, and GenBank accession number for the sequences included in this study.

Table S2 - Number of simple sequence repeats (SSRs) and long repeats present in six Sapotaceae species.

Figure S1 - Comparison of the IR and single-copy regions junctions from six Sapotaceae plastomes.
