# Supplementary material for: The first complete plastome of Mimusops coriacea (A. DC.) Miq. (Sapotaceae)
Source: Genet Mol Biol. 2022 Jan 24;45(1):e20210174. doi: 10.1590/1678-4685-GMB-2021-0174 (PMC8796699; doi:10.1590/1678-4685-GMB-2021-0174)
Supplement: Table S1 - [file 1415-4757-GMB-45-1-e20210174-s1.pdf]

Genetics and Molecular Biology
https://doi.org/10.1590/1678-4685-GMB-2021-0174

Supplementary Material to "The first complete plastome of *Mimusops coriacea* (A. DC.) Miq. (Sapotaceae)"

Table S1 - Species names, family, and GenBank accession number for the sequences included in this study.

Species	Family	Accession Number
Alniphyllum eberhardtii Guillaumin	Styracaceae	NC_031892.1
Ardisia bullata G.H.Huang & G.Hao	Primulaceae	MT505713.1
Argania spinosa (L.) Skeels	Sapotaceae	MK533159.1
Camellia fascicularis Hung T.Chang	Theaceae	MW026668.1
Changiostyrax dolichocarpus (C.J.Qi) Tao Chen	Styracaceae	NC_042252.1
Chrysophyllum cainito L.	Sapotaceae	MT435527.1
Diospyros blancoi A.DC.	Ebenaceae	NC_033502.1
Diospyros hainanensis Merr.	Ebenaceae	NC_042160.1
Diospyros maclurei Merr.	Ebenaceae	NC_042161.1
Lysimachia congestiflora Hemsl.	Primulaceae	NC_045275.1
Madhuca hainanensis Chun & F.C.How	Sapotaceae	MT909828.1
Manilkara zapota (L.) P.Royen	Sapotaceae	MN295595.1
Pouteria caimito Radlk.	Sapotaceae	MT712131.1
Pouteria campechiana (Kunth) Baehni	Sapotaceae	NC_033501.1
Sideroxylon wightianum Hook. & Arn.	Sapotaceae	NC_041130.1
Synsepalum dulcificum (Schumach. & Thonn.) Daniell	Sapotaceae	MT723946.1
