# Supplementary material for: The first complete plastome of Mimusops coriacea (A. DC.) Miq. (Sapotaceae)
Source: Genet Mol Biol. 2022 Jan 24;45(1):e20210174. doi: 10.1590/1678-4685-GMB-2021-0174 (PMC8796699; doi:10.1590/1678-4685-GMB-2021-0174)
Supplement: Table S2 - [file 1415-4757-GMB-45-1-e20210174-s2.pdf]

Genetics and Molecular Biology
https://doi.org/10.1590/1678-4685-GMB-2021-0174

Supplementary Material to "The first complete plastome of *Mimusops coriacea* (A. DC.) Miq. (Sapotaceae)"

Table S2 - Number of simple sequence repeats (SSRs) and long repeats present in six Sapotaceae species.

Species	Microsatellites (SSRs)						Repeats			
	**Mono**	**Di**	**Tri**	**Tetra**	**Penta**	**Total**	**Complementary**	**Forward**	**Palindromic**	**Reverse**
Madhuca hainanensis	293	45	4	6	0	348	8	14	18	10
Manilkara zapota	289	44	2	7	1	343	0	25	13	3
Mimusops coriacea	287	47	4	5	1	344	8	18	15	8
Pouteria caimito	293	46	6	9	1	355	2	13	22	6
Sideroxylon wightianum	292	48	5	8	1	354	6	15	13	16
Synsepalum dulcificum	287	44	7	9	1	348	0	12	13	5
