# Supplementary material for: The first complete plastome of Mimusops coriacea (A. DC.) Miq. (Sapotaceae)
Source: Genet Mol Biol. 2022 Jan 24;45(1):e20210174. doi: 10.1590/1678-4685-GMB-2021-0174 (PMC8796699; doi:10.1590/1678-4685-GMB-2021-0174)
Supplement: Figure S1 - [file 1415-4757-GMB-45-1-e20210174-s3.pdf]

Genetics and Molecular Biology
https://doi.org/10.1590/1678-4685-GMB-2021-0174

Supplementary Material to "The first complete plastome of *Mimusops coriacea* (A. DC.) Miq. (Sapotaceae)"

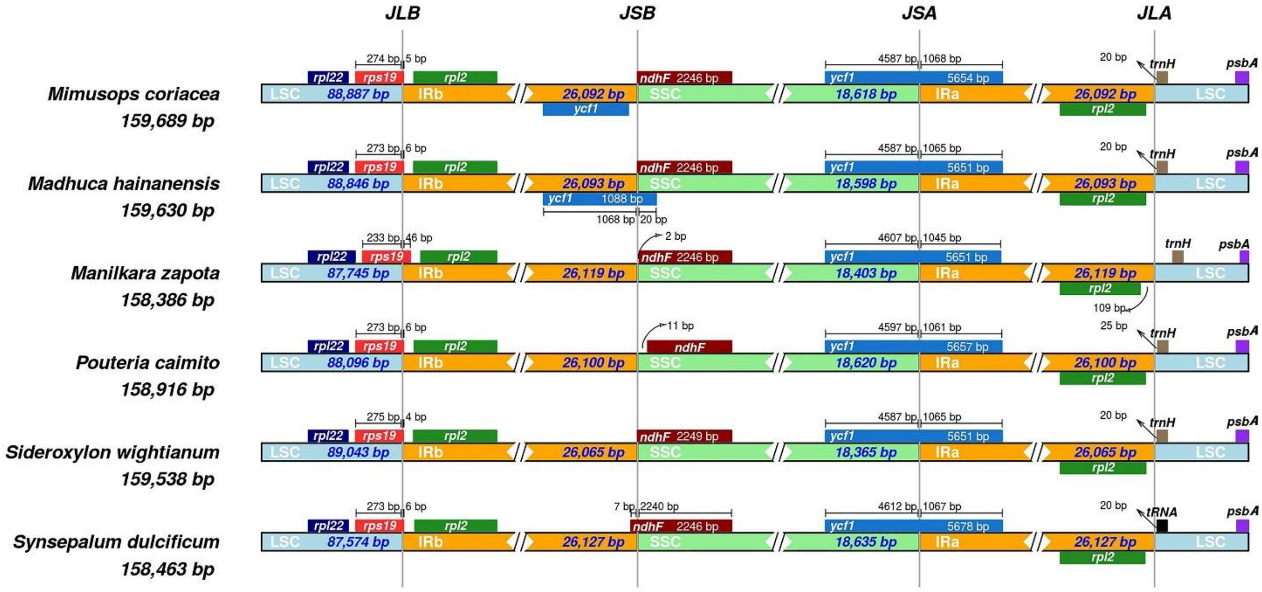

Figure S1 – Comparison of the IR and single-copy regions junctions from six Sapotaceae plastomes. JLB (IRb/LSC), JSB (IRb/SSC), JSA (SSC/IRa) and JLA (IRa/LSC) denote the respective limit in the genome.
